# The Role of Tricellulin in Epithelial Jamming and Unjamming via Segmentation of Tricellular Junctions

**DOI:** 10.1002/advs.202001213

**Published:** 2020-06-08

**Authors:** Sophie Lohmann, Costanza Giampietro, Francesca M. Pramotton, Dunja Al‐Nuaimi, Alessandro Poli, Paolo Maiuri, Dimos Poulikakos, Aldo Ferrari

**Affiliations:** ^1^ Laboratory of Thermodynamics in Emerging Technologies ETH Zurich Zurich 8092 Switzerland; ^2^ EMPA Swiss Federal Laboratories for Materials Science and Technology Experimental Continuum Mechanics Dübendorf 8600 Switzerland; ^3^ IFOM‐ The FIRC Institute of Molecular Oncology Spatiotemporal organization of the nucleus Unit Milan 20139 Italy; ^4^ Institute for Mechanical Systems ETH Zurich Zürich 8092 Switzerland

**Keywords:** cell jamming, epithelial monolayers, tricellular tight junctions, tricellulin, wound healing

## Abstract

Collective cellular behavior in confluent monolayers supports physiological and pathological processes of epithelial development, regeneration, and carcinogenesis. Here, the attainment of a mature and static tissue configuration or the local reactivation of cell motility involve a dynamic regulation of the junctions established between neighboring cells. Tricellular junctions (tTJs), established at vertexes where three cells meet, are ideally located to control cellular shape and coordinate multicellular movements. However, their function in epithelial tissue dynamic remains poorly defined. To investigate the role of tTJs establishment and maturation in the jamming and unjamming transitions of epithelial monolayers, a semi‐automatic image‐processing pipeline is developed and validated enabling the unbiased and spatially resolved determination of the tTJ maturity state based on the localization of fluorescent reporters. The software resolves the variation of tTJ maturity accompanying collective transitions during tissue maturation, wound healing, and upon the adaptation to osmolarity changes. Altogether, this work establishes junctional maturity at tricellular contacts as a novel biological descriptor of collective responses in epithelial monolayers.

## Graphical abstract

A semi‐automatic image processing pipeline is developed to investigate the role of tricellular tight junctions (tTJs) establishment and maturation in jamming and unjamming transitions of epithelial monolayers. The software is used to determine the evolution of tTJ maturity during tissue maturation, wound healing and osmolarity changes, establishing junctional maturity as a novel biological descriptor of collective responses in epithelial monolayers.

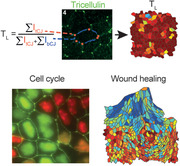

## 1 Introduction

Mature epithelia comprise confluent cohesive sheets of epithelial cells, exerting their function at the interface between the inner and outer compartments of the body.^[^ ^1^ ^]^ The topological organization of polarized epithelial layers enables the biological control of mass transfer during adult homeostasis.^[^ ^2^ ^]^ The actuation of morphological changes during the development of epithelial tissues requires the coordination of cellular activities over space and time. These include proliferation, migration, and shape change that together support the attainment of complex 3D architectures, their maintenance, and adaptation to serve a growing body.^[^ ^3^ ^]^ Alterations of these collective processes link to pathological responses including cancer invasion and asthma.^[^ ^4^, ^5^ ^]^ The coordination of collective cell dynamics emerges from the integration of mechanical and biochemical signals.^[^ ^6^ ^]^ Intercellular adhesions, established between neighboring cells in mature epithelia, are key regulators of the mechanotransduction machinery.^[^ ^7^ ^]^ They enable lateral anchorage, control of mechanical tension in the cellular layer, and long‐distance relay of physical and biological signals between cells and with the basal extracellular matrix.^[^ ^8^ ^]^ A wealth of information exists on how force distribution at intercellular adhesions triggers morphogenetic processes^[^ ^9^ ^]^ involving shape changes,^[^ ^10^, ^11^ ^]^ cell polarization, migration,^[^ ^12^ ^]^ extrusion of cells from a monolayer,^[^ ^13^ ^]^ intercalation, and collective movements.^[^ ^14^ ^]^

Cell‐to‐cell junctions are the main sites of intercellular adhesion. They are linked to contractile cortical actomyosin and thus represent hubs for the generation and integration of mechanical forces across cells and tissues.^[^ ^15^ ^]^ Epithelial cell‐to‐cell contacts comprise the well‐described adherens junctions (AJs) vertically spanning the entire lateral interface between neighboring cells, and the tight junctions (TJs) sealing the apical surface and separating it from the basolateral side.^[^ ^16^ ^]^ The core structural component of the AJs consists of the cadherin/catenin basic adhesive unit.^[^ ^17^ ^]^ Cadherins are transmembrane adhesion receptors that mediate fundamental structural and signaling activities required for adhesion through the association with catenins.^[^ ^18^ ^]^ In addition to bicellular junctions, fully mature epithelia feature specific contact sites in regions where three cells meet. Junctional complexes established in these nodal points are named tricellular tight junctions (tTJs). tTJs, forming where three bicellular TJs converge, have been initially identified for their role in the maintenance of epithelial barrier integrity.^[^ ^19^ ^]^

The molecular components of tTJs are tricellulin and angulins.^[^ ^20^ ^]^ Tricellulin, also known as MARVELD2, is a member of the MARVEL protein family, which additionally includes occludin and MARVELD3.^[^ ^21^ ^]^ In mature tissues, tricellulin strongly localizes at the tTJs, whereas it is excluded from bicellular junctions, yielding a typical punctate pattern.^[^ ^22^ ^]^ Tricellulin is an essential tTJs component, ensuring their proper formation, maintenance, and barrier function both in vivo and in vitro.^[^ ^23^ ^]^ Tricellulin‐deficient mice display a disruption of the strands of intramembrane particles connecting bicellular and tricellular junctions in the inner ear epithelia, which selectively affects the paracellular permeability of ions or small molecules.^[^ ^24^ ^]^ Moreover, tricellulin knockdown alters bicellular TJ morphology and decreases transepithelial electrical resistance in epithelial cell lines.^[^ ^25^ ^]^

Epithelial tissue morphogenesis and emerging collective activities are studied in reconstituted epithelial monolayers in vitro; typically using Madin–Darby canine kidney (MDCK) strains as model system.^[^ ^26^ ^]^ The maturation of epithelia has been described in terms of jamming transitions, whereby collective motion ceases as a function of the local cell density. In jammed monolayers, cells no longer exchange neighbors, allowing for the maturation of cell‐to‐cell junctions.^[^ ^27^ ^]^ The capability of reactivating large‐scale collective movements, that is, the process of unjamming, is, however, retained and can be reactivated in response to wounding,^[^ ^28^ ^]^ or along the transition to pathological maladaptation as in the early stages of cancer.^[^ ^5^, ^29^ ^]^

tTJs have been proposed as important regulators of collective epithelial processes, such as jamming and unjamming transitions. They act as cell shape sensors, driving the orientation of cell division and thus contributing to tissue morphogenesis.^[^ ^30^ ^]^ Cell shape memory is preserved at tricellular points upon mitotic cell rounding, guiding the mitotic spindle orientation along the long axis of the cell.^[^ ^30^ ^]^ Finally, tTJs promote the polarization of actin cytoskeleton and regulate the migration of epithelial collectives during tissue morphogenesis and wound healing.^[^ ^22^ ^]^

Due to the central role of cell‐to‐cell junctions in epithelial development and function, various approaches have been proposed to segment individual cell boundaries from microscopy images of epithelial sheets. The resulting values of cell shape, aspect ratio, area, and density as well the topology of neighbors have proven useful for the description of jamming and unjamming transitions.^[^ ^31^, ^32^ ^]^ Automated detection methods, developed to facilitate the analysis, while improving its robustness, employed algorithms such as watershed or active contour to capture the cell shape from the fluorescent signal of AJs or TJs components.^[^ ^33^, ^34^ ^]^ Recently, Cilla et al. introduced the representation of cells as polygons based on the detection of vertices from the second order derivative of image intensity enabling the extraction of further geometric properties such as cell centroid, width, length, and rotation.^[^ ^35^ ^]^ None of these methods, however, considered the evaluation of tTJs in the description of epithelial collectives.

Here, we present a semiautomatic algorithm for the quantitative evaluation of tTJs maturity in epithelial monolayers. The method is based on the segmentation and geometrical classification of bicellular and tricellular adhesions extracted from the linear distribution of TJs components. Once nodal points are detected, the exclusion of tricellulin from bicellular regions is used to provide a tTJs maturation score for each tricellular node yielding spatially resolved maps of tTJs maturation. The algorithm is validated following the jamming transition of different MDCK strains along the cell cycle, in experiments of wound healing, and upon perturbation of mature monolayers with hypo‐ or hyperosmotic media. Altogether, we establish a novel approach and corresponding tool for the analysis of tricellulin‐mediated junctions in epithelial monolayers. We show that tTJs maturation correlates in space and time with the variation of cell shape and density, accompanying jamming and unjamming transitions. Based on this, we propose a quantitative score of tTJs maturation as a novel functional descriptor of the dynamic state of epithelial tissues completing the analysis of collective phenomena in epithelia.

## 2 Results

### 2.1 Jamming Dynamics in Different MDKC Strains

MDCK cells represent a well‐established model system for the in vitro study of collective behavior in epithelial sheets.^[^ ^36^ ^]^ Different strains of MDCK have been reported, in particular featuring the stable transfection of fluorescent proteins in combination with the plasma membrane (PM) or transported to the nucleus to report for the cell cycle phase.^[^ ^37^, ^38^ ^]^

**Figure 1:**
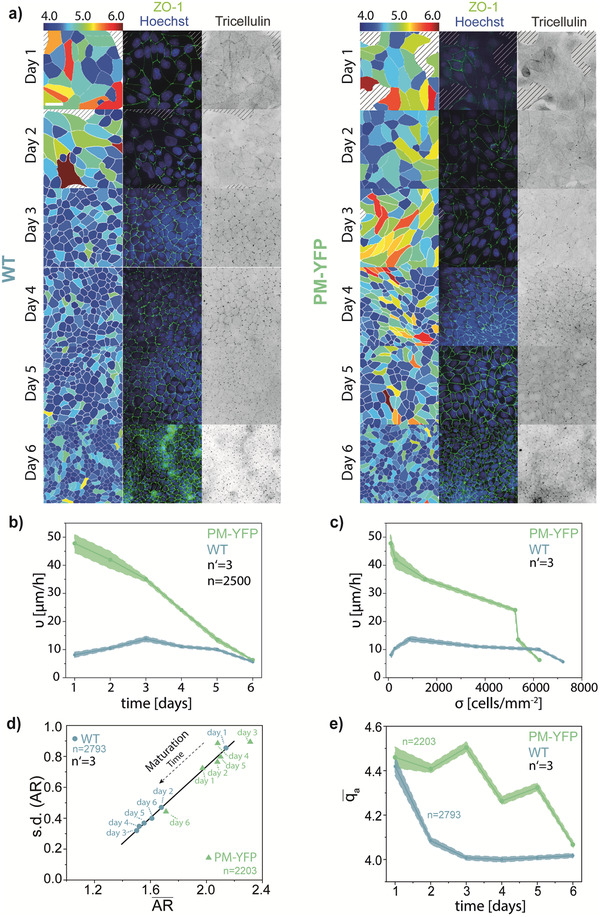
Maturation and jamming behavior of the monolayer of WT and PM‐YFP MDCK cells. a) Representative images of the maturation of the monolayer at days 1–6 of WT and PM‐YFP MDCK cells. Left panels: from the ZO‐1 immunostaining, the image was segmented and cells colored according to the apical shape index (*q*_a_ = *p*_a_/√*A*_a_). A color scale is shown on the top. Middle panels: immunostaining for nuclei (Hoechst, blue) and ZO‐1 (green). Right panels: inverted image of the immunostaining for tricellulin. Scale bar: 50 µm. b) Cell migration velocity, *v*, with respect to time. c) Cell migration velocity, *v*, with respect to cell density *σ*. Shaded areas indicate the standard error of the mean. d) Variation of the mean cell aspect ratio ($\bar{\mathrm{AR}}$) and of its SD (AR) with increasing days of maturation for WT and PM‐YFP MDCK. e) Evolution of the mean apical shape index ($\bar{q_{a}}$) over time.

To calibrate the jamming dynamics as function of time (*T*), cell density (*σ*), and cell shape, we adopted two distinct MDCK type II strains: wild type (WT) MDCK and MDCK stably expressing monomeric yellow fluorescent protein (YFP) at the plasma membrane (PM) (PM‐YFP^[^ ^37^ ^]^). All selected strains form fully mature and polarized epithelial layers in vitro.^[^ ^38^, ^39^ ^]^ In particular, subconfluent cells layers (100 cells mm^–2^) were seeded on fibronectin coated glass slides and imaged along 6 days. In these experimental conditions, the evolution of *σ* and cell migration velocity (*v*) was determined using a cell image velocimetry approach (CIV;^[^ ^40^ ^]^ **Figure** **1**). Epithelial jamming transitions entail a reduction of the initial *v*, which progressively decreases as function of *σ*. Jammed epithelia are recognized by constant cell density and limited cellular movement.^[^ ^12^ ^]^

MDCK strains showed a similar evolution over a period of 6 days (Figure 1a), however, with different dynamics. Specifically, the migration velocity was lower in WT compared to PM‐YFP MDCK cells. Conversely, PM‐YFP cells displayed high *v*, which persisted until the system eventually jammed (Figure 1b–c; Figure S1 and Movies S1 and S2, Supporting Information) at *σ* > 4000 cells mm^–2^.

The establishment of TJs, revealed by the distribution of the marker ZO‐1 in monolayers fixed and immunostained at subsequent times, rendered the development of the apical cell morphology. A continuous network of apical junctions appeared by day 3 in WT monolayers while later, at day 4, in the PM‐YFP MDCK (Figure 1a). Along the same line, a punctate distribution of tricellulin, reporting a mature network of tTJs, arose with a temporal pattern synchronized with the other reporters of epithelial jamming transition.

Cell shape is an independent structural descriptor of the dynamic state in dense epithelial monolayers.^[^ ^41^ ^]^ Immature (i.e., non‐jammed) epithelia typically feature cells with elongated and inhomogeneous shapes. On the other hand, cells are typically smaller and feature a regular (i.e., polygonal) profile in mature, jammed epithelia.^[^ ^31^ ^]^ From the ZO‐1 signal, it was possible to obtain a precise measure of apical membrane perimeter (*p*_a_) and area (*A*_a_) yielding an apical shape index (*q*_a_) defined as $q_{a}=\frac{p_{a}}{\sqrt{A_{a}}}$. The time variation of *q*_a_ (Figure 1d) reports a trend for WT with values converging to a stable plateau by day 3. On the other hand, the mean *q*_a_ in PM‐YFP monolayers remained high until day 5, reaching comparable low levels only by day 6 (Figure 1d).

The convergence of the cell shape and of its cell‐to‐cell variability in jamming systems is conveniently reported by the linear relationship between the aspect ratio (AR) and its standard deviation (SD; (AR)) across the monolayer.^[^ ^31^ ^]^ Upon maturation of the epithelium, both the mean of the AR and the SD (AR) become progressively smaller.^[^ ^31^, ^32^ ^]^ This trend emerges clearly in our experimental models, with WT MDCK attaining converging by day 3 (Figure 1e) and PM‐YPF only later by day 6 (Figure 1e).

### 2.2 Detection of Junctional Topology and Quantitative Measure of tTJ Maturation

The biological maturation of tTJs proceeds through a sequence of stereotyped phases, which has been previously described.^[^ ^23^ ^]^ Once TJs are established at the apical side of neighboring cells in a maturing epithelial monolayer, tricellulin is enriched along their entire span. This diffuse localization progressively converges towards the tricellular edges, geometrically evolving from a 1D pattern to a punctate array (0D).

**Figure 2:**
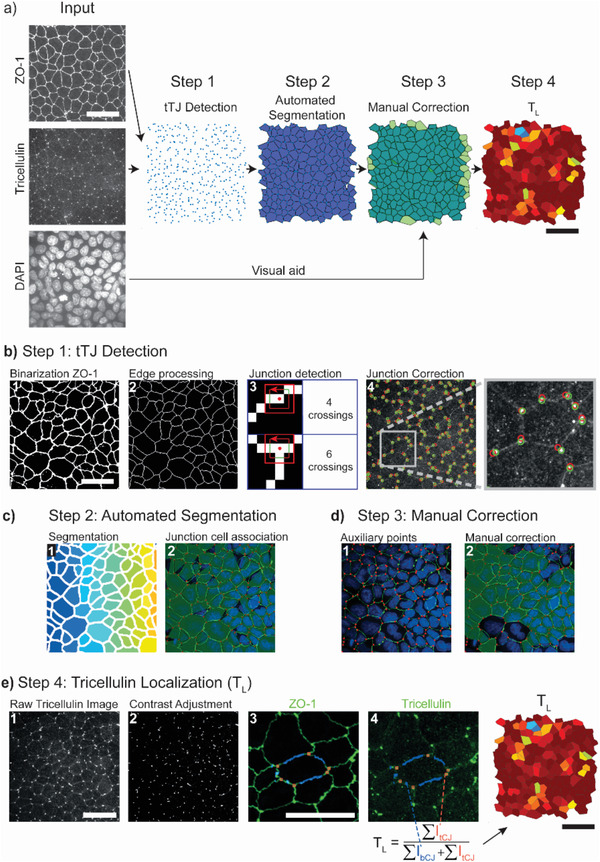
A custom software yielding quantitative measures of tricellular junction maturation. a) Overview of the four steps for tricellulin detection and cell segmentation. b) Step 1: tricellular junction (tTJ) detection: creation of a binary image (1) from the ZO‐1 staining is followed by image processing to reduce edges to single pixel lines (2). A pixel‐counting algorithm (3) is used to determine nodal points where three cells meet. The location of tricellular junctions is corrected (4) using a point source detection algorithm on the raw tricellulin staining. c) Step 2: automatic segmentation: segmentation of the single line edge image (b2) determines pixel regions for each cell (1). Polygonal representations (2) of each cell are created from associated junctions. d) Step 3: manual correction: auxiliary points to improve the shape of cells can be manually added (1) and cell shapes corrected (2). e) Step 4: tricellulin localization: the tricellulin image (1) is contrast adjusted (2). The masks for bicellular junctions (3) are determined from the combination of the cell polygon with the binarized ZO‐1 staining. Tricellulin localization is used as proxy of tTJ maturation (4). It is defined as the ratio of average pixel intensity of tricellular junctions (orange) to that of bicellular junctions (blue). Scale bars: 50 µm.

Based on these observations, we aimed at distilling a parameter providing a reliable quantitative score for the maturation state of tTJs in epithelial monolayers, consisting of four steps (**Figure** **2**). The initial input for the proposed pipeline are three images (Figure 2a) acquired upon fluorescent labeling of the nuclei (e.g., DAPI), of a junctional component of the TJ network (e.g., ZO‐1) and a component of the tricellular junctions (e.g., tricellulin).

The maximal signal projection of the TJ fluorescent Z‐stack renders thin lines, which are detected and binarized in step 1 of the pipeline (Figure 2b). The resulting binary image is processed with a morphological operation to reduce edges to single pixel lines. Identifying crossings of two lines creates a map of vertices and edges, where edges are bicellular adhesions and vertices are tricellular nodes. This topological separation provides a spatially resolved map of the nodal points at which three cells meet, allocated as sites of potential tTJ establishment. To identify the true location of tTJs, vertices are translated to the closest point of high intensity in the tricellulin image. In step 2 (Figure 2c), the junctional image is segmented into individual cells that are linked to tricellular junctions in their proximity. These two initial steps of data analysis are fully automated. However, a degree of user interaction is implemented in the pipeline to ensure the validity of results after visual check. With this purpose, a custom user interface (TricGUI) was designed combining automatic steps with manual corrections (step 3; Figure 2d) in a streamlined manner (see Supporting Information).

The second required experimental input is a fluorescent image reporting the distribution of a tTJ component (e.g., tricellulin) in the same Z region. The resulting immunostaining protocol may render micrographs with low signal‐to‐noise ratio. Therefore, an automated step of background subtraction and contrast adjustment was implemented (see Experimental Section) as part of the pipeline. The map of nodal points, generated from the topological analysis of TJ signal, is now superimposed on the tricellulin signal. In step 4 (Figure 2e), the ratio between the average tricellulin signals measured at a tricellular nodal point and the contiguous bicellular junctions is defined as a measure of tTJs maturity (Figure 2b). Based on this definition, the tricellulin localization parameter (*T*_L_) assumes values between 0 and 1, where low values indicate little accumulation of tricellulin at tricellular nodes (absence of tTJs). Higher values (e.g., closer to 1) indicate a specific enrichment of tricellulin at tTJs, corresponding to a more advanced maturation state.

### 2.3 Validation of the Method in Confluent Monolayers: Jamming and Cell Cycle Phase Affect Tricellulin Localization at tTJs

**Figure 3:**
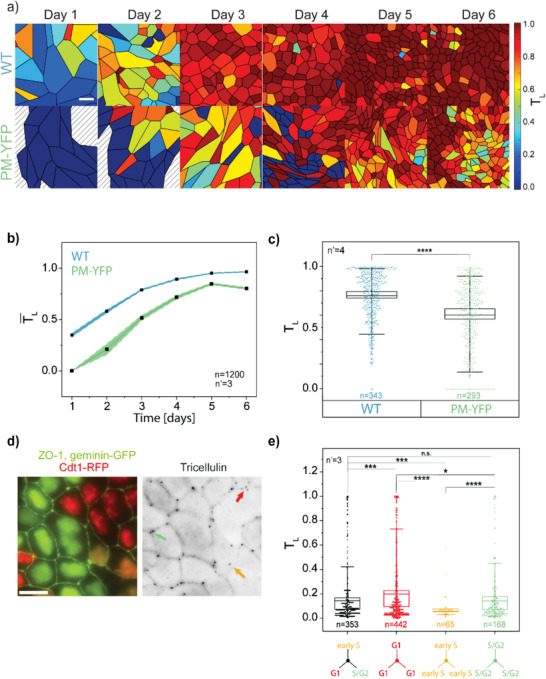
Influence of monolayer maturation and cell cycle phase on tricellulin localization. a) Representative analysis of tricellular junction maturation in WT and PM‐YFP MDCK monolayers from days 1 to 6 obtained using the TricGUI (Figure 2). Cells are colored according to their tricellulin localization index (*T*_L_). A color scale is shown on the right side. Scale bar: 20 µm. b) Comparison of *T*_L_ distribution at tricellular junctions in WT and PM‐YFP MDCK monolayers at day 3. ****p < 10^−6^. c) Evolution of the average *T*_L_ ($\bar{T_{L}}$) from days 1 to 6 for WT and PM‐YFP MDCK cells. Shaded areas indicate the standard error of the mean. d) Representative immunofluorescence image of MDCK‐Fucci monolayer (left) and corresponding tricellulin signal (right). Red, yellow, and green arrows indicate tricellulin junctions (tTj) established by triplets of cells synchronized in G1, early S, or S/G2; respectively. Scale bar: 25 µm. e) Distribution of *T*_L_ values in MDCK‐Fucci cell triplets synchronized in different cell cycle phases (G1, early S, or S/G2) or non‐synchronized. *p < 0.05, ***p < 10^−3^, ****p < 10^−6^.

As first step in the validation of the proposed method, the temporal sequence of epithelial jamming reported in Figure 1 was analyzed (**Figure** **3**). Fluorescent images reporting the distribution of ZO‐1 and tricellulin were collected from WT and PM‐YFP MDCK monolayers at each day of measure (days 1–6). Each field of view contained on average 80 ± 13 cells, corresponding to 420 ± 55 nodal points. The automatic segmentation of individual cell profiles took 24 ± 13 s per image and the full analysis, including manual corrections, was completed in 7:20 ± 2:00 min per image, yielding spatially resolved maps of the tTJs maturation state for each cell in the monolayer (Figure 3a: Movie S3, Supporting Information).

The mean *T*_L_ for the monolayer ($\bar{T_{L}}$), calculated from ≈100 cells per time point, shows that PM‐YFP cells display a reduced level of tTJ maturity as compared to the WT. The time evolution clearly captures the dynamics of jamming in both MDCK strains, showing a delay in PM‐YFP, where the $\bar{T_{L}}$ values are always lower than in the WT (Figure 3b), and well correlated with their sustained motility and slower shape homogenization (Figure 1). This analysis can be extended to a large number of images and cells to resolve subtle differences as indicated by the comparison between the two strains at day 3 (Figure 3c).

In addition, to evaluate the levels of tTJ maturity in different phases of the cell cycle, we used MDCK stably expressing the Fucci2 cell cycle reporting system (MDCK‐Fucci^[^ ^42^ ^]^). The maturation of MDCK‐Fucci monolayers followed a dynamic similar to WT‐MDCK cells (Figure S2, Supporting Information). In particular, the analysis was performed in a confluent monolayer at day 3 selecting tTJs formed by three cells in the same cell cycle phase (Figure 3d). Triplets of cells in the S/G2 phase showed, on average, higher levels of tTJ maturation as compared to triplets in earlier cell cycle phases (G1 and early S), while no significant differences were obtained comparing triplets with cells not synchronized in the same cell cycle phase (Figure 3e). This result is compatible with a partial disassembly of tTJs upon mitosis and their progressive maturation along the cell cycle.^[^ ^20^, ^23^ ^]^

### 2.4 Tricellulin tTJ Localization Correlates with Local Unjamming during Wound Healing

Unjamming transitions can be observed and studied in mature monolayers upon healing of an inflicted wound.^[^ ^36^ ^]^ The process has been thoroughly described^[^ ^28^ ^]^ and entails a partial mesenchymal transitions of cells at the wound edge, forming leaders with enlarged cell body, elongated shape, and active migration.^[^ ^43^ ^]^ In particular, the wound front destabilizes into multicellular migration fingers at the tip of which a leader cell drives the way forward.^[^ ^44^ ^]^ Only the first one to two cells behave as leader cells^[^ ^45^ ^]^ and directional movement must be relayed to the neighboring cells that are induced to follow the leader.^[^ ^46^ ^]^ These settings suggest a concomitant modulation of junctional complexes and, in particular of tTJs, spatially correlating with the reactivation of motility and release of cell shape constrains in a spatial gradient from the tip of the migrating finger to the inner rows of followers.

**Figure 4:**
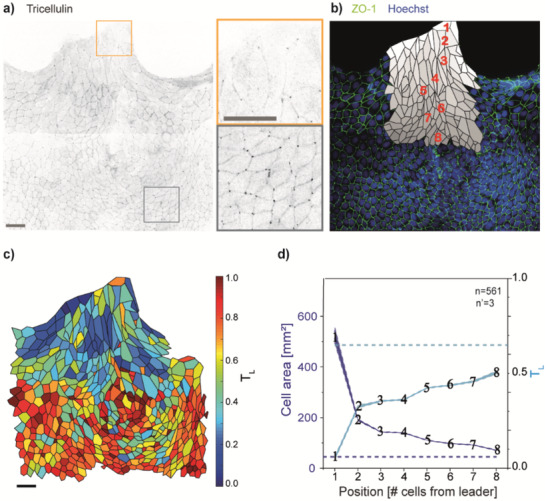
Tricellulin localization in multicellular fingers driving wound healing. a) Inverted fluorescent image of tricellulin signal during wound healing in WT MDCK cells (left). Zoomed view of insets located at the tip of a multicellular finger (green) and in the inner part of the monolayer (red). b) Topological classification of cells in the migration finger. Cells are labeled according to their distance from the leader (i.e., cell 1). c) Tricellulin localization in a representative multicellular migrating finger. A color scale is shown on the right. d) Left axis: mean cell area with respect to the distance from the leader cell. Right axis: tricellulin localization (*T*_L_) with respect to the distance from the leader cell. The dashed lines indicate the mean value for cells in the inner regions of the monolayer (≥15 cells away from the leader cell). Shaded areas indicate the standard error of the mean. Scale bars: 50 µm.

The capability of the proposed software to capture such a transition was challenged in WT MDCK monolayers upon healing of a longitudinal wound (**Figure** **4**). Large fields of view were captured including the developing wound edge and several inner cell rows (Figure 4a). The qualitative analysis of tricellulin junctional localization showed a marked punctate tricellulin distribution deep in the monolayer, which was instead reduced in cells close to the wound edge. To obtain a quantitative description of the cell ensemble, the topological distribution of cells was defined based on their relative position to the wound. In particular, the leader cell was assigned to row 1, its immediate followers to row 2, and this way on till row 8 (Figure 4b).

The quantitative analysis (Figure 2) rendered a spatially resolved map from which a gradient of tTJ maturation can be appreciated (Figure 4c). As shown in Figure 4d, where the spatial distribution of tricellulin localization is plotted along with the cell area, a significant decrease of tTJ assembly level involved the leader cells (*T*_L_ values 0.1). Further away, followers preserved a higher level of tTJ maturity (*T*_L_ values of 0.3–0.5). In particular, a sharp increase in *T*_L_ values between the leader cell and its direct followers (between rows 1 and 2) was found, indicating unique properties of the leader cell itself, which supports previous experimental and theoretical findings of finger cells displaying a mesenchymal phenotype with weaker cell–cell junctions and increased motility.^[^ ^45^, ^47^ ^]^ Follower cells, even at the wound edge, maintain a clear epithelial phenotype, consistent with the establishment of actomyosin contractility.^[^ ^44^ ^]^ The trend of the cell area is anti‐correlated, indicating that the process of shape change and surface enlargement, subtending unjamming, are linked to tTJ disassembly, in agreement to what has been previously reported.^[^ ^30^ ^]^

### 2.5 Active Modulation of tTJs by Osmotic Pressure: Membrane Tension Increases Tricellulin tTJ Localization

**Figure 5:**
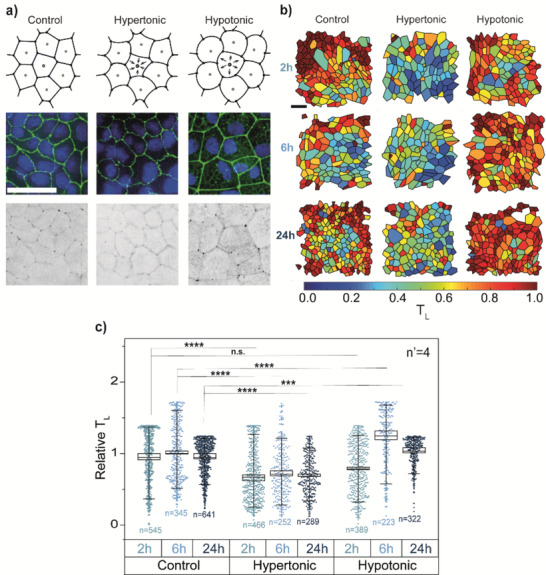
Influence of cell medium osmolarity on tricellulin localization. a) Top row: schematic representation of the effect of cell medium osmolarity on cell volume in WT MDCK monolayers. Middle row: representative combined image of ZO‐1 (green) and nuclei (Hoechst, blue) staining. Bottom row: representative inverted image of tricellulin staining. From left to right: control, hypertonic and hypotonic medium. Scale bar: 50 µm. b) Representative segmentation of cells at 2, 6, and 24 h of treatment. Cells are colored according to their tricellulin localization (*T*_L_). Scale bar: 50 µm. c) *T*_L_ values for 2 h (blue), 6 h (light blue), and 24 h (dark blue) after addition of control (left), hypertonic (middle) or hypotonic medium (right). Values of *T*_L_ are normalized to the control at 6 h. ***p < 10^−3^; ****p < 10^−6^.

The balance of junctional tension between neighboring cells drives the maturation of tTJs at nodal points.^[^ ^23^ ^]^ Membrane and junctional tension, in epithelial monolayers, can be artificially modulated by the variation of osmotic pressure, whereby hypertonic or hypotonic media drive their decrease or increase, respectively.^[^ ^48^ ^]^ It has previously been reported that epithelial cells, in the context of a confluent monolayer, react and adapt mechanically to the exposure to osmotic stress. In particular, to preserve membrane tension homeostasis, cells swell or shrink in response to hypotonic or hypertonic stimulation respectively.^[^ ^48^ ^]^ Interestingly, performing ultrastructure analysis of epithelial monolayers, Nilsson et al. demonstrated that hyperosmotic solution impairs the proper organization of bicellular TJs, thus increasing the permeability of the paracellular pathway.^[^ ^49^ ^]^ Since tTJ establishment and maintenance depend on bicellular TJ proper organization,^[^ ^25^, ^50^ ^]^ we next tested whether the response to osmotic variations entails an alteration of tTJ assembly exposing mature epithelia to hyper or hypotonic media and following their effect over time at 2, 6, and 24 h (**Figure** **5**). A qualitative observation of junctional assembly (i.e., the ZO‐1 distribution) confirmed an effect on bicellular junctions, with increased ruffling in the hypertonic condition and straightened adhesions in the hypotonic one, which correlated with changes in membrane tension.^[^ ^48^ ^]^ At the same time, tricellulin distribution showed qualitative variations, with more intense localization at the nodal points in hypotonic media (Figure 5a).

These subtle variations were well‐captured by the software analysis (Figure 5b). The maps of tTJ maturation state rendered a clear overview of the overall increase in hypotonic media and of a moderate decrease in hypertonic ones. These visual representations were translated in quantitative plots (Figure 5c), which display the statistically significant increase of tTJ maturity in hypotonic media, peaking at 6 h post‐treatment. The cell response to hypertonic condition was more rapid, being already significant 2 h post‐treatment, and showed a sustained decrease of tricellulin at tTJs, in agreement the impairment of bicellular TJ organization.^[^ ^49^ ^]^ These results suggest that tricellulin is recruited at tTJs in response to increased tension, as previously reported for vinculin at tricellular AJs.^[^ ^23^, ^51^ ^]^ Moreover, a differential kinetics of tTJ maturation in response to hypertonic or hypotonic stimulation is identified. Overall, the proposed software analysis allowed capturing the time‐dependent modulation of tTJ assembly accompanying the response of epithelial monolayer to variation of osmotic pressure and tension.

### 2.6 Composition of AJs in MDCK Strain with Delayed Jamming

Jamming and unjamming transitions in epithelia are linked to cell shape adaptation.^[^ ^31^, ^52^ ^]^ In this frame, many factors are responsible or contribute to shape the cell profile, including cell cycle progression, apoptosis, and cadherin‐dependent adhesions (AJs) to the neighbors.^[^ ^4^ ^]^ The latter, in particular, are essential to generate coherent sheets of epithelial cells with distinct apical‐basal polarity.^[^ ^53^ ^]^ In a mature epithelium, each cell displays an apical and a basolateral membrane domain, differing in their membrane protein composition.^[^ ^54^ ^]^ AJs serve as landmark to separate these two membrane domains. The cell shape is also strongly influenced by AJs through tension established by junctional cadherin.^[^ ^55^ ^]^

**Figure 6:**
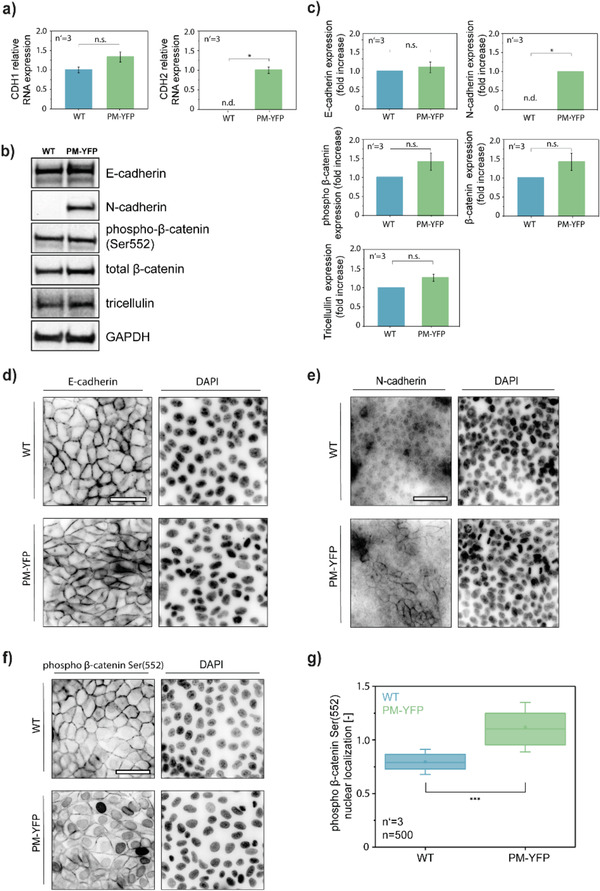
Differences in junctional organization between MDCK WT and PM‐YFP. a) qRT‐PCR analysis for the expression of CDH1 (E‐cadherin) and CDH2 (N‐cadherin) in MDCK WT and PM‐YFP. *p < 0.05. b) WB analysis of junctional proteins and c) relative quantification. *p < 0.05. d, e) Immunofluorescence analysis of E‐cadherin and N‐cadherin in MDCK WT and PM‐YFP. f) Immunofluorescence analysis of phospho‐*β*‐catenin (Ser552) and g) relative quantification. ***p < 0.001. Scale bars: 50 µm.

To identify the mechanism responsible for the delayed jamming transition in PM‐YFP MDCK cells, we analyzed the composition of AJ complexes (**Figure** **6**). qRT‐PCR, Western blot (WB), and immunofluorescence analysis revealed no variation in the expression level and localization of epithelial (E)‐cadherin. However, PM‐YFP MDCK showed high levels of neuronal (N)‐cadherin, localized at cell‐to‐cell contacts (Figure 6a–e). Importantly, E‐cadherin‐based AJs are significantly more stable than those established by N‐cadherin.^[^ ^56^ ^]^ N‐cadherin is typically expressed by cells with mesenchymal phenotype such as fibroblasts and overexpressed in several types of cancer where it drives detachment from the primary tumor, invasion, and metastatization.^[^ ^57^ ^]^ This similarity suggests that AJs established by PM‐YFP MDCK fail to provide sufficient mechanical stability, as normally ensured by E‐cadherin, thus delaying the jamming transition.

To gain further evidence, we focused on *β*‐catenin signaling, a crucial trigger of cell motility in epithelial cells^[^ ^58^ ^]^ and a core component of the AJ complex.^[^ ^18^ ^]^ Phosphorylation of the Ser552 residue induces *β*‐catenin disassociation from cell–cell contacts and the translocation into the nucleus where its transcriptional activity is exerted.^[^ ^59^, ^60^, ^61^ ^]^ WB analysis performed on total cell lysates did not show any difference between WT and PM‐YFP MDCK (Figure 6b–c). On the other hand, immunofluorescence revealed a strong nuclear phospho‐*β*‐catenin signal in PM‐YFP MDCK, as compared to a junctional localization in the WT counterpart (Figure 6f–g). Given the fundamental role of E‐cadherin, N‐cadherin, and *β*‐catenin signaling in epithelial cell proliferation and motility,^[^ ^16^ ^]^ these results suggest that differences in junctional complexes composition impact on the collective movement of WT and PM‐YFP MDCK cells.

## 3 Discussion and Conclusion

We have developed and validated an image analysis software providing a spatially resolved quantification of the maturation state of tTJs. The software pipeline requires experimental information reporting the distribution of bicellular junctions, a biological prerequisite for the establishment of tTJs, which allows for the segmentation of neighboring cells and supports the detection of nodal points where three cells meet.

The categorization of junctional sites into bicellular or tricellular contacts defines the location where tTJs can be formed, that is, the points in the monolayer where three cells converge. The tTJ maturation score is then distilled based on the actual distribution of one of its main components, that is, tricellulin. Along the maturation of tTJs, tricellulin is initially enriched at bicellular junctions and gradually concentrated at nodal points in a process of maturation that requires the establishment of a balance of forces among the cell triplet.^[^ ^23^ ^]^ Leveraging on this biological mechanism, the tTJ maturation state is measured as the exclusion of tricellulin signal from sites of bicellular contact, implying the accumulation at vertexes.

The robustness and level of automation of the analysis pipeline depend of the quality of the original images, and therefore on the specificity of the fluorescent reporters labeling the bicellular and tricellular junctions. Here, ZO‐1 and tricellulin were selected as markers of TJs and tTJs, respectively. However, the application of the analysis is not restricted to these proteins and can be extended to other equivalent junctional components. The possibility of manual correction is included in the protocol, as to correct for local errors due to imaging or labeling artifacts. Again, the level of manual intervention depends on the original quality of the experimental data.

The tTJ maturation parameter was obtained along the maturation of MDCK monolayers providing resolved maps to illustrate the composition and evolution of cell ensembles. Large number of values, informing of the tTJs established by individual cells, can be extracted and statistically compared. When this analysis was performed on different MDCK strains, it resolved variations of jamming dynamics, suggesting a direct involvement of junctional maturation in the reduction of migration velocity with increasing cell density. Alternative jamming mechanisms have been reported, such as the progressive decrease of traction forces transmitted to the substrate at the level of lamellipodia.^[^ ^52^ ^]^ However, the characteristic composition of cell‐to‐cell junctions in MDCK strains with delayed jamming (PM‐YFP) supports the hypothesis that junctional instability demotes epithelial maturation and enhances cell motility in densely packed monolayers. These results are in agreement with physical models demonstrating the dominant role of junctional assembly in slowing down cell motility upon maturation of epithelial monolayers.^[^ ^27^ ^]^

Even more compelling is the detection of parametric variations within the same monolayer upon wound healing. The mapped spatial distribution closely underlines the cell shape variation typical of mesenchymal transition in multicellular migrating fingers at the wound front. The analysis of tTJs offers a clear representation of the monolayer remodeling from the leader cell to the inner followers, and implies a role of tTJs in the regenerative response leading to local unjamming and reactivation of directional migration. Within this context, the software is able to capture the effect of environmental perturbations know to impinge in the balance of junctional forces. Osmotic changes affect the maturation state of tTJs, as revealed by the quantitative analysis, with a dynamic response compatible with a role of tTJs in the regulation of jamming and unjamming transitions.

Altogether, our proposed software provides an easy access to the quantitative evaluation of tTJs in epithelial monolayers with a tunable level of automation. The resulting parameter represents a proxy measurement for the maturation state of epithelial tissues capturing the junctional aspects regulating cell collective behavior.

## 4 Experimental Section

##### Cell Culture and Treatments

MDCK WT and PM‐YFP cells^[^ ^37^, ^38^ ^]^ were cultured in Dulbecco's modified Eagle's medium‐high glucose media (Sigma‐Aldrich) supplemented with 10% fetal bovine serum (FBS, Sigma‐Aldrich), 100 U mL^−1^ PenStrep (LifeScience), and 2 × 10^−3^ m L‐glutamine (LifeScience). MDCK‐Fucci^[^ ^40^, ^42^ ^]^ were cultured in MEM (1×)+GlutaMAX‐I supplemented with 10% FBS and 100 U mL^−1^ PenStrep. All cells were maintained at 37 °C and 5% CO_2_. In all epithelialization experiments, a total of 10^4^ cells cm^–2^ cells were seeded on glass substrates.

##### Antibodies

The following primary antibodies were used: rabbit monoclonal MARVELD2 antibody (IF: 1:100; 54H19L38, Thermo Fisher Scientific), mouse monoclonal ZO‐1 antibody, Alexa Fluor 488 or 647 (IF: 1:100; ZO1‐1A12, Thermo Fisher Scientific), mouse monoclonal E‐cadherin antibody (IF: 1:100, WB: 1:1000; 610181, BD Biosciences), mouse monoclonal N‐cadherin antibody (IF: 1:250, WB: 1:2500; 561553, BD Biosciences), rabbit monoclonal phospho‐*β*‐catenin (Ser552) (IF:1:100, WB: 1:1000; 9566, Cell signaling).

Secondary antibodies were chicken anti‐rabbit Alexa 647 (1:400, Thermo Fisher Scientific, A‐21443), donkey anti‐mouse Alexa 555 (1:400, Thermo Fisher Scientific, A‐32773), anti‐rabbit IgG HRP‐linked (1:2000, Cell Signaling, 7074), anti‐mouse IgG HRP‐linked (1:2000, Cell Signaling, 7076).

##### Western Blotting

Confluent cells were lysed by boiling in a modified Laemmli sample buffer (2% sodium dodecyl sulfate, 20% glycerol, and 125 × 10^−3^ m Tris‐HCl, pH 6.8). Equal amounts of proteins were loaded on gels, separated by sodium dodecyl sulfate‐polyacrylamide gel electrophoresis, and transferred to a nitrocellulose membrane (Protran; Whatman). After incubation with primary and HRP‐linked secondary antibodies, specific bindings were detected by a chemiluminescence system (GE Healthcare).

##### Immunostaining

Cells were fixed for 20 min with 4% paraformaldehyde at room temperature or with ice‐cold methanol at 4 °C. Next, the cells were permeabilized with 0.5% Triton X‐100 in phosphate‐buffered saline (PBS) for 5 min. Afterwards, they were incubated in 5% w/v bovine serum albumin (Sigma‐Aldrich, USA) in PBS for 1 h at room temperature. The samples were incubated with the respective primary antibodies (see section Antibodies) overnight at 4 °C.

Subsequently, the samples were rinsed three times for 5 min with PBS. They were then incubated with the corresponding secondary antibody for 1 h at room temperature. Finally, the samples were washed three times for 5 min with PBS. For staining of nuclei, Hoechst was added at 10 µg mL^−1^ during a washing step.

##### Cell Microscopy

Cell movement was monitored using an inverted Nikon‐Ti wide‐field microscope (Nikon, Japan) and an incubation chamber (Life Imaging Services, Switzerland). The medium was maintained at a controlled temperature of 37 °C and CO_2_ concentration of 5%. Images were collected with a 20×, 0.45 NA long‐distance objective (Plan Fluor, Nikon, Japan). Time‐lapse experiments were set to routinely collect images, in different spatial positions of the sample, in the BF channel with a time resolution of 20 min.

ZO‐1, tricellulin, EDU‐stained nuclei, and Hoechst or DAPI stained nuclei distribution were acquired in immunostained samples using a 60×, 1.4 NA oil immersion objective (Plan Fluor, Nikon, Japan), and the FITC, Cy5, TRITC, and DAPI filter respectively. Samples were images with an inverted Nikon‐Ti spinning disk confocal microscope (Nikon, Japan) equipped with an Andor DU‐888 camera (Oxford Instruments, UK) and a pE‐100 LED illumination system (CoolLED Ltd., Andover, UK).

##### Proliferation Assay

The DNA synthesis‐based cell proliferation assay was performed using a commercially available Click‐iT EdU Imaging Kits Protocol (Thermo Fisher Scientific) and following the manufacturer recommendations. Cells were seeded on the substrates and incubated for 24 h with 10 × 10^−6^ m 5‐ethynyl‐2ʹ‐deoxyuridine (EdU) labeling solution before fixation (days 1, 2, 3, 4, and 5). After fixation with 4% formaldehyde and permeabilization with 0.5% Triton X‐100 in PBS, samples were stained and imaged using a fluorescence microscope.

##### Wound Healing Assay

Confluent cell monolayers were wounded by manually scratching with a pipette tip, washed with fresh medium to remove cell debris, and incubated with fresh media for 11 h. Afterwards wounded samples were fixed and immunostained.

##### Osmotic Treatment

For PM tension perturbation by osmotic treatments, an equal volume of hypotonic buffer (H_2_O + 1 × 10^−3^ m CaCl_2_ + 1 × 10^−3^ m MgCl_2_) or hypertonic buffer (complete growth medium+200 × 10^−3^ m sucrose‐containing complete growth medium; final concentration 100  × 10^−3^ m) was added to the cells when they reached confluence at 2 to 3 days after culture. Cells were incubated for 2, 6, or 24 h before fixation and immunostaining.

##### Statistical Analysis

The two sample, two‐sided Kolmogorov–Smirnov test was used to test the null hypothesis that both samples were taken from the same distribution, as it is sensitive to both shape and location of the distribution function. Boxes in all box plots indicate the standard error of the mean, with a line at the median and a square representing the mean. Whiskers extend to the 10th and 90th percentile. The total number of events counted is reported as *n* in the graphs. All results were confirmed in at least three independent experiments.

##### Image Processing

To ensure validity of results, image processing was largely avoided for quantifications, apart from a projection and contrast adjustment step. Because cell monolayers do not have a constant thickness, Z‐stacks were acquired during confocal microscopy. To exclude out of plane signal in tricellulin images, instead of computing a maximum projection, the ZO‐1 staining was used to determine the z‐layer of highest intensity for 20 × 20 pixel blocks. The tricellulin image was then composed of the equivalent z‐layers for 20 × 20 patches. For the resulting projection of the tricellulin image, image contrast was adjusted in an automated fashion. Since there is no tricellulin in the nucleus, image noise was computed by using the nuclear staining as a mask to compute the average pixel intensity of the tricellulin image in the nuclear area. To compute the signal‐to‐noise ratio, the maximum pixel intensity was determined: $\mathrm{SNR}=\sqrt{\frac{\mathrm{Max}\;\mathrm{Intensity}}{2\;\times \;\mathrm{Noise}}}$. The tricellulin image was then adjusted by subtracting the measured noise level from all pixels and then dividing by the calculated signal‐to‐noise ratio.

##### Image Analysis

A custom user interface was implemented in MATLAB for the semi‐automatic analysis of fluorescence microscopy images of immunostaining. Detailed information on the workflow and algorithms of this software can be found in Supporting Information.

##### Velocity Measurement

Cell image velocimetry (CIV) toolbox was used for the analysis of the wound healing time series as previously described.^[^ ^40^ ^]^ The velocity field provided by the CIV analysis was quantified by the mean cell layer speed (mean velocity magnitude) along 32 equally spaced sectors.

Tracking measurements were performed using the cell tracking software Imaris (Bitplane Scientific Software, Switzerland). Time‐lapse videos were uploaded into Imaris and the velocity and density of the cells were obtained by tracking the migration of individual cells over time until confluence.

##### qRT‐PCR Analysis

RNA was extracted from MDCK cells (WT and PM‐YFP) using silica columns. In brief, cells were lysed in 350 µL Solution D (4 M guanidine thiocyanate, 25 × 10^−3^ m sodium citrate, 0. 5% Sarkosyl containing freshly added *β*‐mercaptoethanol (350 × 10^−3^ m) to inhibit RNAse activity) and 350 µL of 70% EtOH was added and the lysate was loaded directly on mini‐spin silica column. Column was centrifuged for 1 min at 10 000 rcf, then washed with 450 µL of 4 M sodium acetate. Next, two washes with 350 µL of 70% EtOH were performed. Column was dried by 2 min of centrifugation, and RNA was eluted with 50 µL of RNase free water. RNA amount was analyzed by Nanodrop and cDNA was synthesized with a Retrotranscription Kit (Life Technologies) following manufacturer protocol. qRT‐PCR was then performed using TaqMan Gene Expression Master Mix (Thermo Fisher). Genes and probes used for the study are enlisted here after: CLDN2 Cf02712471_g1, CDH1 Cf02697525_m1, CDH2 Cf02696085_m1, and GAPDH Cf04419463_gH. Results were normalized on to the level of the ubiquitously expressed GAPDH and were expressed as 2^−ΔΔCt^ (normalized on the WT sample).

## Conflict of Interest

The authors declare no conflict of interest.

## Supporting information

Supporting Information

Supplemental Movie 1

Supplemental Movie 2

Supplemental Movie 3
