# Supplementary material for: The Role of Tricellulin in Epithelial Jamming and Unjamming via Segmentation of Tricellular Junctions
Source: Adv Sci (Weinh). 2020 Jun 8;7(15):2001213. doi: 10.1002/advs.202001213 (PMC7404176; doi:10.1002/advs.202001213)
Supplement: Supplementary file 1 — Supporting Information [file ADVS-7-2001213-s001.pdf]

© 2020 Wiley-VCH GmbH

ADVANCED SCIENCE
Open Access

Supporting Information

for *Adv. Sci.*, DOI: 10.1002/advs.202001213

On the Role of Tricellulin in Epithelial Jamming and Unjamming via Segmentation of Tricellular Junctions

Sophie Lohmann[1,#], Costanza Giampietro[2,#], Francesca M. Pramotton[1,#], Dunja Al-Nuaimi[1], Alessandro Poli[3], Paolo Maiuri[3], Dimos Poulikakos[1,], and Aldo Ferrari[1,2,4,*]*

On the Role of Tricellulin in Epithelial Jamming and Unjamming via Segmentation of Tricellular Junctions

TricGUI: Supporting Information

Sophie Lohmann[1,#], Costanza Giampietro[2,#], Francesca M. Pramotton[1#,], Dunja Al-Nuaimi[1], Alessandro Poli[3], Paolo Maiuri[3], Dimos Poulikakos[1,*], and Aldo Ferrari[1,2,4,*].

1. ETH Zurich, Laboratory of Thermodynamics in Emerging Technologies, 8092 Zurich, Switzerland
2. EMPA, Swiss Federal Laboratories for Materials Science and Technology, 8600 Dübendorf, Switzerland
3. IFOM, The FIRC Institute of Molecular Oncology, 20139 Milan, Italy
4. ETH Zurich, Institute for Mechanical Systems, 8092 Zürich, Switzerland

[#] These authors contributed equally to the work

[*] Corresponding authors

SUPPLEMENTARY METHODS

This document provides a detailed description of the semi-automatic workflow to extract tricellulin localization, as well as cell area and cell shape from fluorescence microscopy images of immunostainings.

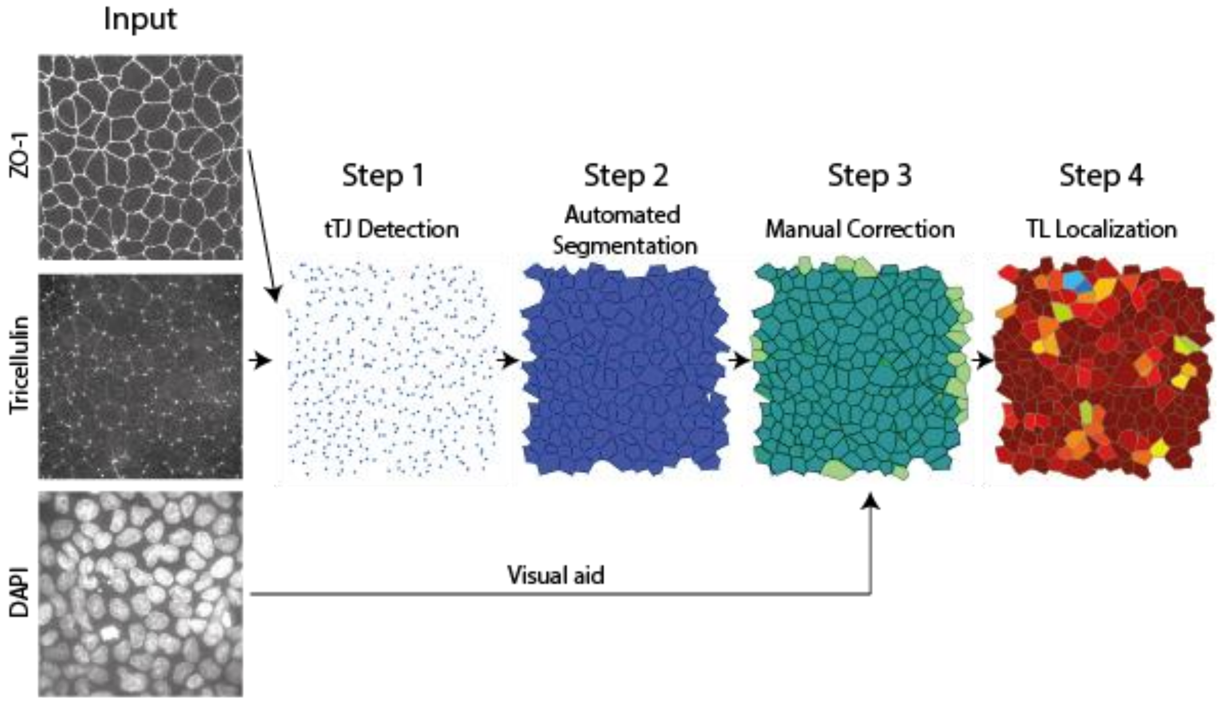

Figure SM1: The 4 steps to semi-automatically determine tricellulin localization to tTJs from images of ZO-1, tricellulin and DAPI stainings.

TricGUI uses three fluorescence microscopy images as input. All data is extracted from the tricellulin staining. The ZO-1 staining is used for junction and cell detection, as well as junction mask definition to be applied to the tricellulin staining. The nuclei staining solely improves the visualization for manual user input.

1. Tricellular tight junction (tTJ) detection

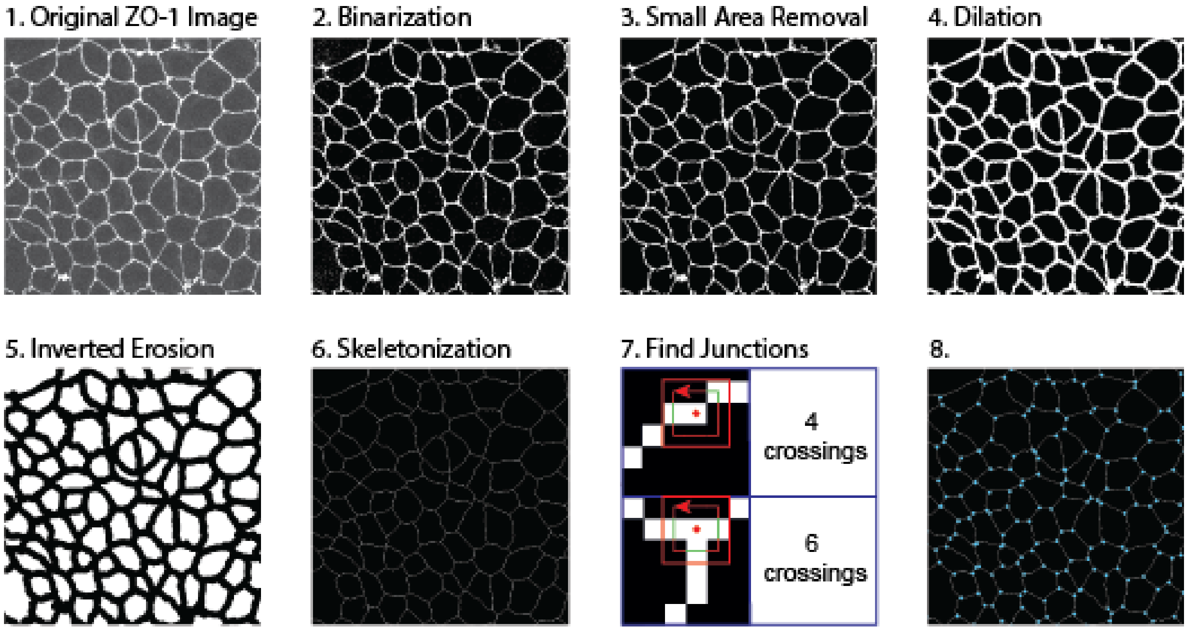

Figure SM2: Automatic tTJ detection from a fluorescence microscopy image of a bicellular junction staining (here ZO-1).

Primary tTJ detection. The position of tricellular junctions is first detected using cell shapes extracted from a bicellular junction staining image (here ZO-1) and refined using a tTJ staining image (tricellulin).

The pipeline for primary detection of tricellular junctions from the ZO-1 image is illustrated in Figure SM2 and can be described as follows:

1. First, the raw, grayscale ZO-1 image (Figure SM2 (1))…
2. …is binarized by applying an adaptive threshold computed by Bradley's method[1] (Figure SM2 (2)). This means that the binarization threshold is variable for each pixel of the image, determined by the local mean intensity in its neighborhood.

3. All connected components (areas of white pixels) that consist of fewer than 20 pixels are removed (set to zero/black) (Figure SM2 (3)).
4. The binary image is then dilated using a disk with a 2 pixel radius as a structuring element before removing connected components consisting of fewer than 100 pixels (Figure SM2 (4)).
5. To remove potential small holes in junctions, the image is inverted before removing connected components smaller than 200 pixels. An erosion step using a disk structuring element with a 1 pixel diameter follows (this is equivalent to dilating the inverted image) (Figure SM2 (5)).
6. After inverting again so that bicellular junctions are positive, a morphological operation[2] removes white pixels until it has shrunk bicellular junctions to minimally connected strokes or single pixel lines. Then, spur pixels that are only connected to other pixels by a corner are also removed (Figure SM2 (6)).
7. In the resulting one-dimensional white lines, a pixel is considered a node (tricellular junction), if the number of crossings between 0 and 1, i.e. black and white, on the perimeter of its 3x3 neighborhood is 6 or 8 (Figure SM2 (7)).
8. This allows to determine the position of tricellular junctions (Figure SM2 (8)).

tTJ refinement. While the primary detection results in a close approximation of the tricellular junction location, it often fails to locate the exact position of the maximum tricellulin signal. Therefore, the tricellulin image is used to refine the exact position of tTJs as displayed in Figure SM3 and described in the following four steps:

1. In the grayscale tricellulin image, a segment of 15x15 pixels around each node determined from the ZO-1 image is selected (Figure SM3 (1)).

2. The selected 15x15 pixel image segment is padded by replicating the border pixel 7 times, resulting in a 29x29 pixel images The node position is then corrected by least-square fitting a 2D Gaussian to tricellulin image segments of 15x15 pixels around each node. The exact algorithm is described by Lendenmann et al[3]. The best fit improves detecting the position of the highest source of tricellulin signal of each tricellular junctions (Figure SM3 (2)).
3. This node correction is repeated for each previously detected node (Figure SM3 (3)).
4. Furthermore, junctions in very close to vicinity to another (Euclidean distance smaller than 5 pixels) are removed (Figure SM3 (4)).

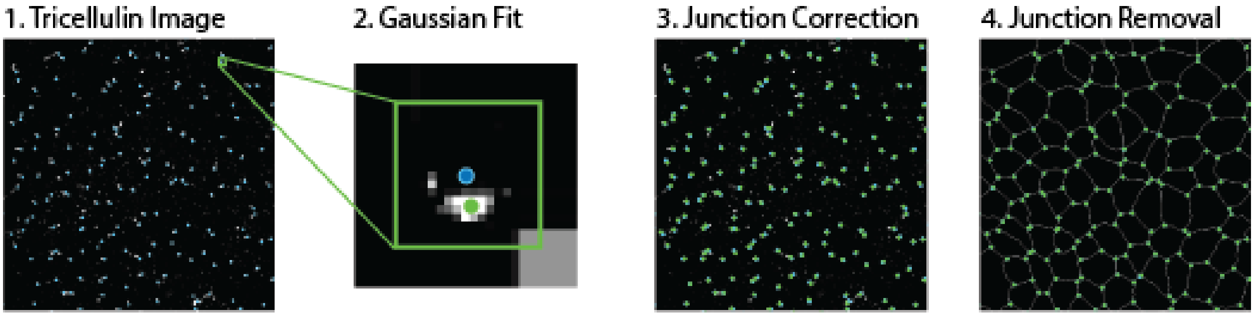

Figure SM3: Refinement of tTJ position from the tricellulin staining image.

2. Segmentation of cells

To calculate tricellulin localization from tricellulin intensity and to extract information on cell shape and size, cells are approximated as polygons defined by the cells' tTJs. For this purpose, the binary junction image of single pixel strokes (Figure SM2 (6)) is inverted.

1. Connected components below a size of 400 pixels and above a size of 60,000 pixels, as well as connected components that touch the border are removed (Figure SM4 (1)).
2. Each connected region is then considered one cell. Each cell is linked to its tricellular junctions. For this purpose, a range search algorithm uses a Kd-tree[4] to determine the indices of nodes whose Euclidean distance is less than 10 pixels to any pixel coordinates of

the connected region defined as a cell. This means that any node less than 10 pixels away from a cell is considered a tricellular junction of that cell (Figure SM4 (2)).

3. Any junction that is not linked to a cell is discarded (Figure SM4 (3)).
4. For each cell, the nodes linked to that cell are considered vertices of a polygon that approximates the cell shape (Figure SM4 (4)).

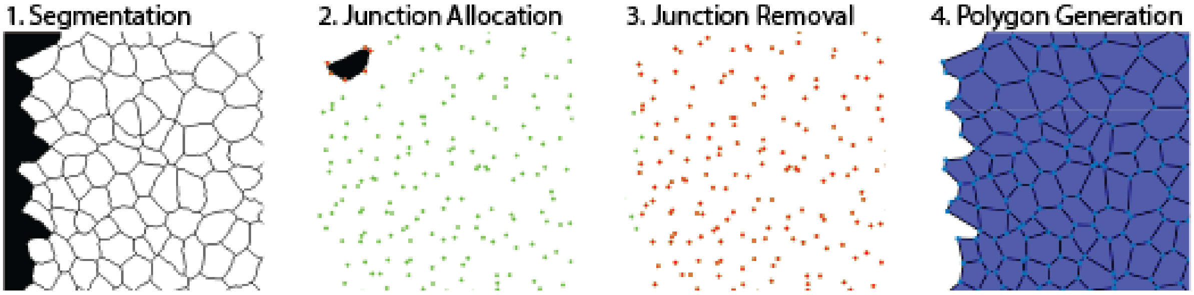

Figure SM4: Generation of polygons to approximate cells with tTJs as vertices.

3. Manual correction cells

Each cell then visualized as a polygon defined by its tTJ on top of a montage of ZO-1 and DAPI images on the left-hand side of the GUI as well as the tricellulin image on the right-hand side of the GUI.

Depending on the quality of the ZO-1 staining, it can happen that the algorithm connects two or more cells, places tTJs on artefacts from bicellular junctions, misses one of its tTJs or includes a different, close tTJ. Therefore, the GUI allows correcting, deleting and adding polygons as well as junctions.

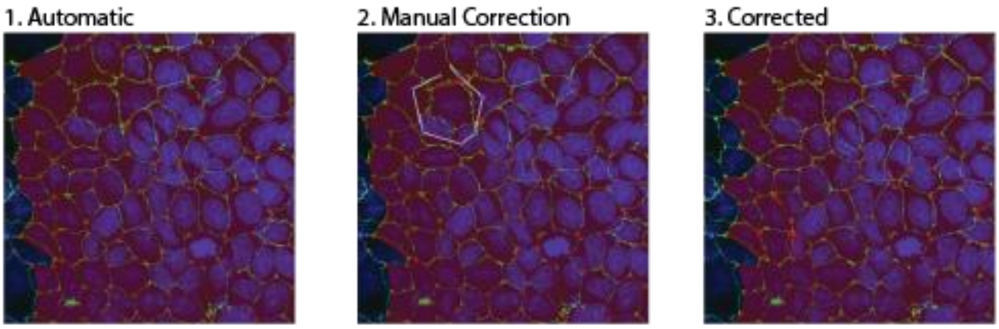

Figure SM5: Manual correction of cell shapes.

Junctions:

- Add Junctions: after clicking the “add junctions” button or pressing “a”, the user can click on as many positions in the image (tricellulin or ZO-1) as desired until pressing “Enter”. The selected positions are then refined analogously to the automatic correction in Section 1 (Figure SM3 (1-3)).
- Delete Junctions: after clicking the “delete junctions” button or pressing “d”, the user can click close to as many nodes as desired until pressing “Enter”. A KD-tree is then used to search for the node coordinates closest (Euclidean distance) to each click coordinate. These coordinates are then discarded. Nodes that are already part of a polygon cannot be discarded.
- Move junctions: after pressing “m”, the user can click close to a node and then click a second time to indicate the corrected position of the selected node. A KD-tree is used to find the closest node (Euclidean distance) to the first click. The position of the second click is refined analogously to the automatic correction in Section 1. In order to correct the polygons that the corrected node is a part of, the algorithm loops through all polygons and checks whether the selected node is part of it. If so, the polygon is updated with the corrected junction position.

Polygons:

- Add polygons: to add polygons, the user can click on “new cell” or press “n” to draw a polygon or click on “new cell rect” or press “x” to draw a rectangle. The latter requires less clicks, making it faster, but fails in areas with many junctions. The algorithm then loops through all nodes and determines whether they fall inside the drawn shape. If so, they are considered part of the new cell. After looping through all nodes, a new polygon is generated, if three or more nodes where found to be inside the drawn shape.
- Delete polygons: to delete polygons, the user can press “9” and click on all the polygons to be deleted until pressing “Enter”. For each click, the algorithm loops through all polygons and checks, whether the click coordinates are inside that polygon. If so, the polygon is discarded.
- Edit polygons: if the user knows the number of the polygon to be edited, e.g. if it was the last polygon edited, the user can select the number from a drop down menu. Alternatively, the user can click on “Edit Polygon” or press “k”, then click on the polygon to be edited in the image and draw a new polygon as if adding a new polygon. The algorithm loops through all the polygons and checks whether the click is inside that polygon. This polygon is then replaced by the polygon generated analogously to adding a new polygon.

4. Tricellulin Localization

Tricellulin localization is defined as the ratio of the summed tricellulin signal from tTJ pixels to the sum of signal from tricellular and bicellular junctions pixels.

$$T_L = \frac{\sum I_{tCJ}}{\sum I_{bCJ} + \sum I_{tCJ}}$$

For each cell, the tTJ pixels are defined as 3x3 pixel squares around the tricellular junctions linked to that cell. I_{tCJ} denotes the greyscale value of the pixel that is part of a tricellular junction. $\sum I_{tCJ}$ denotes the sum of the greyscale values of all pixels within the 3x3 squares around the tricellular junctions of a cell. I_{bCJ} denotes the greyscale value of a bicellular junction pixel. The pipeline for identifying bicellular junction pixels is illustrated in Figure SM6 and can be described as follows:

1. The ZO-1 image is binarized using an adaptive threshold determined by Bradley's method[1] (Figure SM6 (1)).
2. Loop over all cells:
   a. The polygon shape of the current cell scaled by the factor 1.3 and converted to a mask of the same size as the tricellulin image, so that pixels inside the polyon are 1 and outside 0. This mask is multiplied with the binarized ZO-1 image (Figure SM6 (2)).
   b. This results in a mask consisting of only the bicellular junctions of the current cell (Figure SM6 (3)).
   c. A binary image of the same size as the tricellulin image is generated so that all pixels are set to 1 except for 10x10 pixel boxes around each tricellular junction of the current cell that are set to 0. This image is then multiplied with the bicellular junction mask that resulted from the previous step (Figure SM6 (4)).
   d. The multiplication results in a mask in the shape of the bicellular junction of the current cell excluding the tricellular junctions (Figure SM6 (5)).

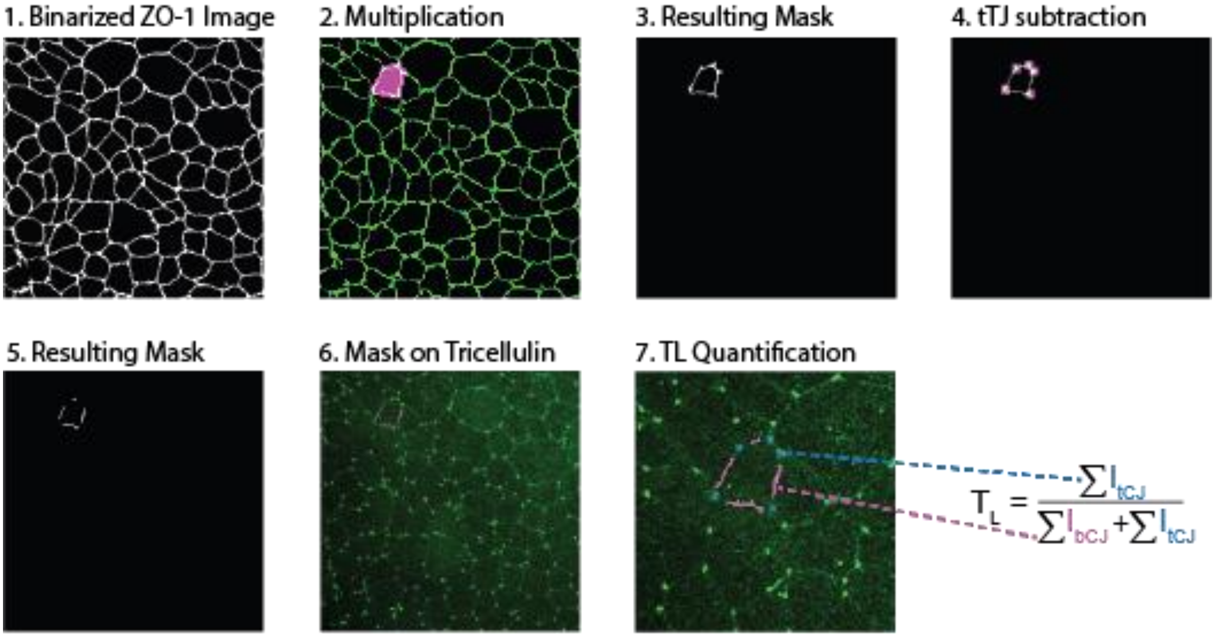

Figure SM6: Mask generation for tricellulin localization quantification.

5. Additional Functionalities

Cell labeling

For wound healing and cell division experiments, cells from the same field of view need to be categorized. Cells can therefore be labeled with a numeric value. The numeric value in the form of a positive integer can be entered in the respective text box. Alternatively, pressing "i" increases the value by 1 and pressing "o" decreases the value by 1. Then, the user can press "c" before clicking on all the polygons to be labeled with the current label and confirm with enter. For each click, the algorithms loops through all polygons and determines whether the position of the click is within the polygon. If so, the current label is assigned to that polygon. New polygons are automatically assigned the current label which is 1 if left unchanged after starting TricGUI.

Visualization

Pressing ‘p’ visualizes the polygons on top of the ZO-1 image on the left and on top of the tricellulin image on the right. The polygon transparency is set to 0.3 (0 being fully transparent and 1 fully opaque). Cell polygons are colored according to their label. Starting at 1, the colors red, orange, yellow, green, blue and purple are periodically repeated (Figure SM7).

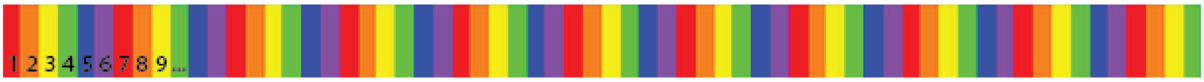

Figure SM7: Cell label colors.

Alternatively, cell can be visualized on top of the ZO-1 and tricellulin images according to different values using the colormap in Figure SM8:

Tricellulin Localizaton: Pressing “1” calls the algorithm described in 4 and subsequently plots cell polygons according to their tricellulin localization between 0 and 1.

Area: Pressing “2” plots polygons colored according to their area with values ranging from 200 μm^2 to 1000 μm^2.

Shape Factor: Pressing “3” computes the shape factor $q = \frac{\sqrt{Perimeter}}{Area}$ and plots polygons colored accordingly with values ranging from 3 to 6.

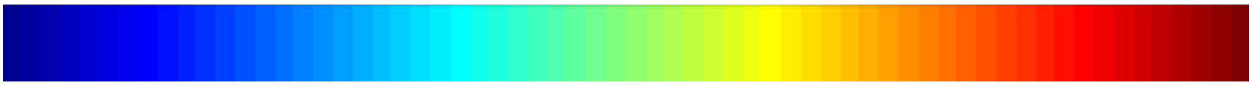

Figure SM8: Colormap for visualizing cell tricellulin localization, area and shape factor.

Heatmap

In addition to visualizing tricellulin localization as cell properties, pressing ‘7’ runs an algorithm for calculating tricellulin localization per junction and displays it as a heatmap. The masking algorithm is analogous to that described in section 4. However, instead of using the

polygon shape to generate the bicellular junction mask, a 30x30 pixel box around each junction is used. This allows to determine the specific tricellulin localization of each junction.

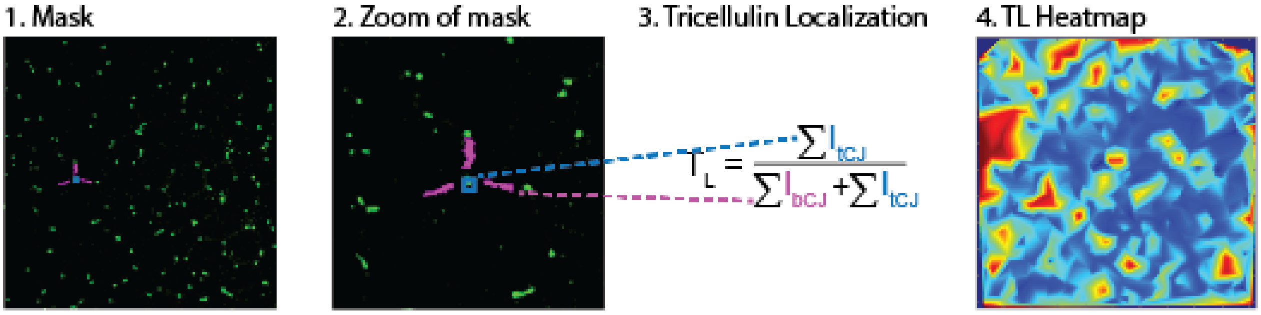

Figure SM9: Colormap for visualizing cell tricellulin localization, area and shape factor.

SUPPLEMENTARY FIGURES

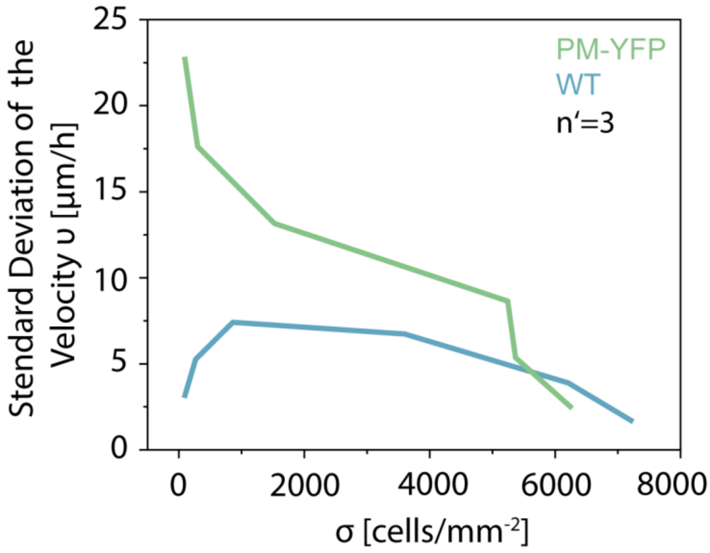

Supplementary Figure 1: Evolution of the standard deviation of the cell migration υ with respect to cell density σ for WT and PM-YFP MDCK.

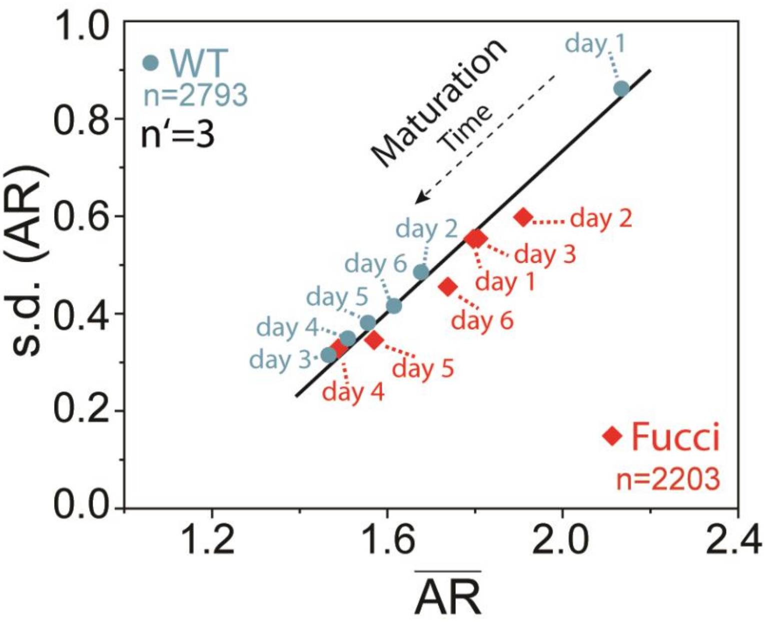

Supplementary Figure 2: Variation of the mean cell aspect ratio ($\overline{AR}$) and of its standard deviation (s.d.(AR)) with increasing days of maturation for WT and MDCK-Fucci.

Supplementary Movie 1: Video of transmission microscopy time-lapse of MDCK WT cells during 120 hours of culture.

Supplementary Movie 2: Video of transmission microscopy time-lapse of MDCK PM-YFP cells during 120 hours of culture.

Supplementary Movie 3: Screen recording of a representative analysis obtained using TricGUI.

SUPPLEMENTARY REFERENCES

1. Bradley, D. and G. Roth, *Adaptive Thresholding using the Integral Image.* Journal of Graphics Tools, 2011. **12**(2): p. 13-21.
2. Lam, L., S.W. Lee, and C.Y. Suen, *Thinning methodologies-a comprehensive survey.* IEEE Transactions on Pattern Analysis and Machine Intelligence, 1992. **14**(9): p. 869-885.
3. Lendenmann, T., et al., *Cellogram: On-the-Fly Traction Force Microscopy.* Nano Lett, 2019. **19**(10): p. 6742-6750.
4. Freidman, J.H., J.L. Bentley, and R.A. Finkel, *An Algorithm for Finding Best Matches in Logarithmic Expected Time.* ACM Transactions on Mathematical Software, 1977. **3**(3): p. 209-226.
